# Sustained remission after haploidentical bone marrow transplantation in a child with refractory systemic juvenile idiopathic arthritis

**DOI:** 10.1186/s12969-021-00523-3

**Published:** 2021-03-12

**Authors:** Guillaume Morelle, Martin Castelle, Graziella Pinto, Sylvain Breton, Matthieu Bendavid, Charlotte Boussard, Richard Mouy, Brigitte Bader-Meunier, Michaela Semeraro, Albert Faye, Marina Cavazzana, Bénédicte Neven, Stéphane Blanche, Pierre Quartier, Despina Moshous

**Affiliations:** 1 Pediatric Hematology-Immunology and Rheumatology Department, Necker-Enfants-Malades University Hospital, Assistance Publique Hôpitaux de Paris, 149 rue de Sèvres, 75015 Paris, France; 2 Pediatric Endocrinology Department, Necker-Enfants-Malades University Hospital, Assistance Publique Hôpitaux de Paris, Paris, France; 3 Pediatric Radiology Department, Necker-Enfants-Malades University Hospital, Assistance Publique Hôpitaux de Paris, Paris, France; 4 Imagine Institute, Université de Paris, Paris, France; 5 Reference Center for Rheumatic, AutoImmune and Systemic diseases in children (RAISE), Paris, France; 6 Université de Paris, Paris, France; 7 Clinical Research Centre, Necker-Enfants-Malades University Hospital, Assistance Publique Hôpitaux de Paris, Paris, France; 8 Pediatric Department, Robert Debre University Hospital, Assistance Publique Hôpitaux de Paris, Paris, France; 9 Gene and Cellular Therapy Unit, Necker-Enfants-Malades University Hospital, Assistance Publique Hôpitaux de Paris, Paris, France

**Keywords:** Systemic juvenile idiopathic arthritis, Still’s disease, Allogenic hematopoietic stem cell transplantation, Graft versus host disease, Autoimmunity

## Abstract

**Background:**

Some patients with systemic juvenile idiopathic arthritis (SJIA) and severe, refractory disease achieved remission through intensive immunosuppressive treatment followed by autologous hematopoietic stem cell transplantation (HSCT). However, disease relapsed in most cases. More recently selected SJIA patients received allogenic HSCT from a HLA-identical sibling or a HLA matched unrelated donor. While most transplanted patients achieved sustained SJIA remission off-treatment, the procedure-related morbidity was high.

**Case report:**

A girl presented SJIA with a severe disease course since the age of 15 months. She was refractory to the combination of methotrexate and steroids to anti-interleukin (IL)-1, then anti-IL-6, tumor necrosis factor alpha inhibitors, and thalidomide. Given the high disease burden and important treatment-related toxicity the indication for a haploidentical HSCT from her mother was validated, as no HLA matched donor was available. The patient received a T replete bone marrow graft at the age of 3.7 years. Conditioning regimen contained Rituximab, Alemtuzumab, Busulfan, and Fludarabine. Cyclophosphamide at D + 3 and + 4 post HSCT was used for graft-versus-host-disease prophylaxis, followed by Cyclosporin A and Mycophenolate Mofetil. Post HSCT complications included severe infections, grade 3 intestinal graft-versus-host-disease, autoimmune thyroiditis, and immune thrombocytopenia. Three years after HSCT, the child is alive and well, notwithstanding persistent hypothyroidy requiring substitution. Immune thrombocytopenia had resolved. Most importantly, SJIA was in complete remission, off immunosuppressive drugs.

**Conclusion:**

Allogenic HSCT may be a therapeutic option, even with a HLA haplo-identical alternative donor, in patients with inflammatory diseases such as SJIA. Despite increased experience with this treatment, the risk of life-threatening complications restrains its indication to selected patients with severe, refractory disease.

## Background

Systemic juvenile idiopathic arthritis (SJIA), as well as Adult Onset Still’s disease, are rare diseases of unknown origin characterized by the association of autoinflammatory features, a risk of macrophage activation syndrome and, in most cases, erosive polyarthritis [1]. Over the last 15 years, new treatments, particularly interleukin (IL)-1 and − 6 inhibitors have shown efficacy to control SJIA activity in most patients thereby avoiding long-lasting steroid-dependency [2–5]. However, some patients do not respond well to biologic treatments and may develop life-threatening complications, including severe lung disease [6, 7]. Intensive immunosuppressive therapy followed by autologous hematopoietic stem cell transplantation (HSCT) enabled difficult-to-treat patients to achieve steroid-free remission [8, 9]. However, some patients developed severe complications including infection-induced macrophage activation syndrome, and a disease flare occurred in most cases within the following years [10–12]. More recently, a few patients were successfully treated by intensive immunosuppression followed by allogenic HSCT with an Human Leucocyte Antigen (HLA)-identical or HLA-matched unrelated donor for SJIA [13] or for severe autoimmune disease [14, 15]. Here, we report a three-year-old girl who was successfully treated with haplo-identical allogenic HSCT.

## Case description

The patient, a girl, is the second child from third degree consanguineous parents. Her older brother is healthy. Besides a vitiligo in the mother, there was no significant family history. At the age of 15 months she developed typical SJIA features including marked, spiking fever, skin rash, polyarthritis of the hips, knees, ankles, elbows, wrists, metacarpo-phalangeal and proximal interphalangeal joints. Assessment for differential diagnoses, including large infectious screening was negative. A diagnosis of SJIA was made. The association of consanguinity, early-onset and severity of the disease suggested an inherited predisposition, but genetic analyzes including whole exome sequencing in the child and her parents did not reveal any validated genetic variant.

**Fig. 1:**
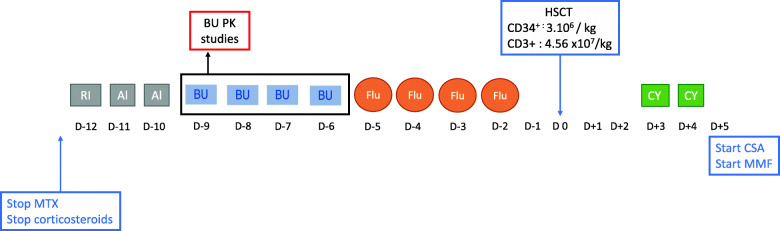
Conditioning regimen. Shows the patient’s conditioning regimen prior to HSCT. BU: Busulfan. Ri: Rituximab. Al: Alemtuzumab. Flu: Fludarabine. CY: Cyclophosphamide. HSCT: hematopoietic stem cell transplantation

At diagnosis, the child received pulsed methylprednisolone followed by daily prednisolone (2 mg/kg per day) in association with indomethacin. Each time prednisolone was gradually tapered to a daily dose of 1 mg/kg, the disease flared. Combination therapy with methotrexate and tocilizumab, then anakinra, canakinumab and adalimumab did not significantly reduce steroid-dependency. Also, an association of methotrexate, thalidomide and infliximab failed to diminish the daily prednisolone dose below 1 mg/kg. Growth retardation, systemic hypertension, osteopenia, and polyarticular erosions (Fig. 1) developed over the following 2 years.

As the patient had severely impaired quality of life as well as treatment related toxicity due to the high dose steroid dependency, there was a clear indication to seek for other treatment options. Allogenic HSCT was considered. In the absence of any possible HLA matched donor, a multidisciplinary team assessed risks and benefits of an alternative graft procedure. Given the high disease burden and treatment related toxicity, the indication for a haploidentical HSCT from her mother was validated. Both parents gave their written informed consent for the procedure, which was approved by the ethics committee of our institution.

At conditioning regimen onset, the child was 3.7 years old. Her weight was 15.6 kg (+ 0.8 SD) for a size of 92.5 cm (− 1.1 SD). She had a cushingoid appearance, arterial hypertension, persistent active painful arthritis of both knees, ankles, wrists and fingers, persistent biologic inflammation with elevated ESR and CRP. Ongoing treatment by methotrexate (5 mg/week), thalidomide (50 mg/day), indomethacin (25 mg/day), and infliximab (75 mg/4 weeks) was stopped shortly before initiating the conditioning regimen. Prednisolone, administered at 1 mg/kg/day prior to conditioning, was gradually tapered and ultimately stopped during the conditioning regimen.

The conditioning regimen (Fig. 1) consisted of rituximab (375 mg/m^2^), alemtuzumab (0.5 mg/kg), fludarabine (160 mg/m^2^), and busulfan with an AUC of 25,638 microM/min. Prophylaxis against graft-versus-host disease (GVHD) contained cyclophosphamide 50 mg/kg at Day 3 and Day 4, as well as cyclosporin and mycophenolate mofetil starting on Day 5. The patient received 3 × 10^6^ CD34^+^ cells/kg from her donor. Stem cell source was bone marrow. During the aplasia, which lasted 24 days, *Actinomyces odonlyticus* sepsis required antibiotherapy with a favorable outcome. Hematologic recovery was achieved on Day 25, with 100% donor chimerism.

Several complications occurred in the post-HSCT course. Grade II acute cutaneous GVHD developed at Day 45 and required steroid therapy; grade III gastrointestinal GVHD developed at Day 53 and was treated successfully with steroids and infliximab. GVHD gradually improved and steroid therapy was tapered and finally stopped 6 months after HSCT. *Clostridium difficile* colitis was diagnosed at Day 59, and the child recovered on antibiotic therapy. Persistent hepatic cytolysis led to an extensive infectious and autoimmune screening that remained negative. Mild nodular hyperplasia was found on a liver biopsy performed at month 11 post HSCT. At the last follow up, liver enzymes were normal (AST 38 UI/l, ALT 13 UI/l).

One year after HSCT, the child developed a clinically asymptomatic autoimmune thrombocytopenia. At this occasion, ongoing intravenous immunoglobulin substitution every 3 weeks was increased temporarily to 1 g/kg. Twenty-three months after HSCT, impaired lung function tests led to a suspicion of obliterating bronchiolitis. However, abnormalities gradually resolved over several months on fluticasone inhalations and oral azithromycin. Two years and 4 months after HSCT, asymptomatic autoimmune thyroiditis was diagnosed upon increased Thyroid stimulating hormone (TSH), positive anti-thyroglobulin, anti-thyroperoxydase and anti-TSH antibodies. L-thyroxin treatment was started. Two years and 9 months after HSCT, the patient presented shingles on the right half of the hemiface with involvement of the ophthalmic (V1) and maxillary branch (V2) of the trigeminal nerve due to varicella-zoster virus reactivation. She had received acyclovir prophylaxis during HSCT until Day 60 according to institutional recommendations. Of note, she had not been vaccinated against VZV. She was treated with IV acyclovir and antibiotics because of a bacterial superinfection (staphylococcal cellulitis). Outcome was favorable.

**Fig. 2:**
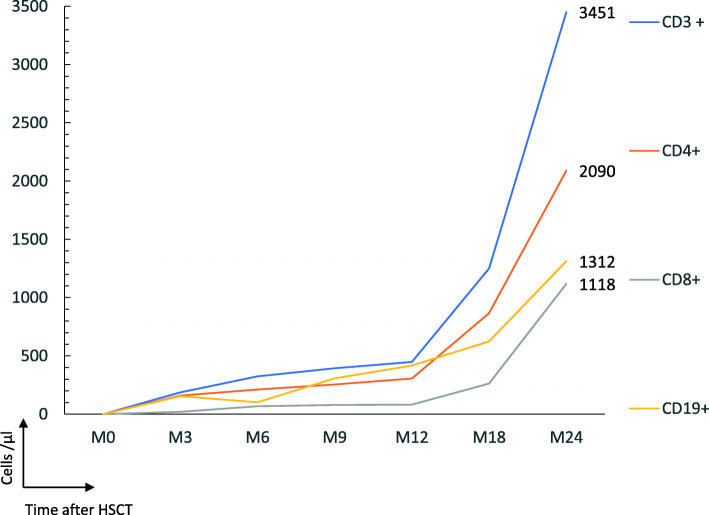
Immune reconstitution after transplantation. Shows the patient’s T- and B-cell reconstitution after transplantation

A transient knee effusion appeared 6 months after HSCT that resolved rapidly without any specific treatment, there was no more evidence of SJIA activity afterwards. At the last follow up, 3.1 years after HSCT, the child was in complete remission of SJIA off steroids and immunosuppressive drugs. She had a regular growth between the 25th and 50th percentile, the weight was at 75th percentile. Clinical examination was normal besides a limited vitiligo at the right upper thorax (situated the previous insertion of the central line), and some white hair strands. Asymptomatic thrombocytopenia had regressed, and the patient was again on weekly subcutaneous immunoglobulin substitution (4 g/week). Platelet count was almost normal (123 G/l, normal values for reticulated platelets). Asymptomatic hypothyroidism required L-Thyroxin treatment. She was also still on 12.5 mg/m^2^/day hydrocortisone substitution for secondary adrenal insufficiency due to previous glucocorticoid therapy and oracillin prophylaxis. Immunoglobulin substitution was stopped, and she had received all recommended post HSCT vaccinations including live vaccines. Immune reconstitution is described in Table 1 and Fig. 2.

**Table 1:** Immune reconstitution after transplantation

Time after HSCT	M + 3	M + 4	M + 5	M + 6	M + 9	M + 12	M + 18	M + 24	M + 36	Normal values in healthy children
Age (years)	3.8	46	47	48	51	54	60	66	78	2 to 6
CD3^+^ (cells/ μl)	186	346	197	324	394	447	1249	3451	4635	1400–3700
CD4^+^ (cells/ μl) - CD31^+^ CD45RA^+^ naive (%) -CD45RO^+^ memory (%)	158	278	131	211	254	305 24 68	866	2090 62 32	2754	700–2200 43–55 58–70
CD8^+^ (cells/ μl)	20	56	36	68	79	81	262	1118	1411	490–1300
-CD45RA^+^CCR7^+^ naive (%)						55		41		52–68
-CD45RA^−^CCR7^+^ central memory (%)						11		1,5		3–4
-CD45RA^−^CCR7^−^ effector memory (%)						28		4,5		11–20
-CD45RA^+^CCR7^−^ TEMRA (%)						6,5		13		16–28
CD19^+^ (cells/ μl)	154	208	55	102	307	417	624	1312	1948	390–1400
CD16^+^ 56^+^ (cells/ μl)	57	125	102	125	149	132	121	49	134	130–720
T cell proliferations (cpm/min/10^3)										
-PHA, J3								72		> 50

*PHA* phytohemagglutinin

The patient achieved a CD4^+^ count > 400/μl at M4. Naïve T cells appeared at M12. The percentage was 24% for CD4 ^+^CD31^+^CD45RA^+^ and 54% for CD8 ^+^CD45RA^+^CCR7^+^

## Discussion

To the best of our knowledge, this is the first report of successful haploidentical HSCT in a child with systemic juvenile idiopathic arthritis.

The procedure was very effective in controlling SJIA activity within a few months, similarly as for most patients previously treated by intensive immunosuppressive conditioning regimens followed by autologous HSCT [16] or allogenic HSCT from HLA-matched related or unrelated donors [13] (Table 2). The achievement of long-lasting, complete SJIA remission off-steroids or other immunosuppressive therapy after successful allogenic HSCT offers hope of definitive cure, which is specific to this peculiar therapeutic procedure. However, a longer follow-up is required. In the largest series of allogenic HSCT in SJIA published so far, 16 patients received HSCT either from an HLA identical sibling or a 9/10 or 10/10 HLA matched unrelated donor. Out of these 16 patients fourteen were alive at the latest follow-up with a median follow-up of 29 months. Importantly, eleven of them were in complete remission off immunosuppressive treatments.

**Table 2:** Results and outcome depending on the type of transplantation^a^

Authors	Brinkman et al.	Abinum et al.	Silva et al.
Year of publication	2007	2009	2017
Number of patients	22	7	16
Location	Netherlands	UK	UK
**HLA donor**	Autologous stem cell transplantation (with T cell depletion)	Autologous stem cell transplantation (with T cell depletion)	MUD^b^ 9/16 MSD: 4/16 mMUD: 3/16
**Age at HSCT (median)**	9.1 years (range 4.2 to 18.2 years)	10.8 years (range 6 to 18 years)	21.5 months (range, 5 months to 12 years)
**Follow up (median)**	70 months (range 13–135 months)	80 months (range 60–96 months)	29 months (range, 2.8–8 years)
**Deaths**	2^d^	1^e^	2^f^
**Complete clinical remission**	8/20	4/6	11/14
**Partial responders**	7/20	0/6	1/14
**Relapse of the disease**	5/20	2/6	1/14
**Infections:**	19/22	4/7	10/16
**Viral**^c^ **(CMV, HSV, EBV, VZV, BK virus)**	25	6	10
**Bacterial**	8	NC	3
**Fungal**	2	NC	1
**Allo/auto-immune complications**	–	1/6	4/14
**GVHD (grade II-IV)**	–	–	3/16

^a^ Two other cases report of autologous hematopoietic stem cell transplantation

- Mistric et al. (*Vntir Lek, 1999*) which was a success

- Quartier.P et al. (*Lancet, 1999*), the child died from toxoplasma infection

^b^*MUD* Matched Unrelated Donor, *MSD* Matched Sibling Donor, *mMUD* Mismatched Unrelated Donor

^c^ *HSV* herpes simplex virus, *CMV* cytomegalovirus, *EBV* Epstein-Barr virus, *VZV* Varicella zoster virus

^d^ Two children died from Macrophage Activation Syndrome (P1 at D + 18, P2 at D + 120)

^e^ One patient died 4 months post-transplant from disseminated adenovirus reactivation

^f^ One child died from invasive fungal infection and one from sepsis twenty months after HSCT

An obvious caveat of allogenic HSCT is the risk of severe complications. Autoimmunity developed in our patient as in others following HSCT. It is unclear whether this was linked to the patient’s genetic background, to the fact that her donor had an autoimmune disease (vitiligo), or to post HSCT dysimmunity in the setting of T replete haploidentical graft procedure. As expected, patients with a long-lasting history of systemic inflammation and corticosteroid therapy cumulate a high risk of infections, drug-induced toxicity and, peculiar to allogenic BMT, GVHD. In the series published by Juliana M. F. Silva et al [13], there were 2 procedure-related deaths among 16 patients, many severe infections, including viral infections favored by intensive immunosuppressive therapy, and 3 cases of grade II to IV graft-versus-host-disease [13]. As there are more and more innovative therapeutic options in SJIA, as witnessed by several currently ongoing trials with new biologics and Janus Kinase inhibitors, only a tiny minority of SJIA patients should be proposed allogenic HSCT. However, the feasibility and safety of this therapeutic approach is also regularly improving, thanks to the increasing experience of haploidentical HSCT in other non-malignant diseases, particularly in patients with primary immunodeficiencies [16].

## Conclusion

SJIA may in some patients have a severe disease course and an inappropriate response to steroids and currently available medications, including biologics. Allogenic HSCT with an HLA-identical sibling or an unrelated HLA-matched donor has been successfully performed in a few patients. We show here that allogenic HSCT with a haplo-identical donor is also feasible in patients with severe, refractory SJIA, and seems to offer long-lasting, if not definitive cure. However, this procedure should remain exceptional in this indication as it is associated with a high risk of complications.

## Data Availability

Please contact authors for data requests.

## Abbreviations

CRP: C-reactive protein
ESR: Erythrocyte sedimentation rate
GVHD: Graft-versus-host disease
HLA: Human leucocyte antigen
HSCT: Hematopoietic stem cell transplantation
IL: Interleukin
SD: Standard deviation
SJIA: Systemic juvenile idiopathic arthritis
TSH: Thyroid stimulating hormone
